# Brighton and Its Three Climates, &c.

**Published:** 1843-07-01

**Authors:** 


Brighton and its Three Climates, &c. &c. &c. By *A. L. Wigan*, M.D. Octavo, pp. 71. Highley, 1843.

London and Brighton have, of late, stretched forth towards each other the right hand of fellowship, with great alacrity. They are now distant from one another some twenty-five miles, and when the flying steamer shall have completely fledged its wings and fleeced its subscribers, the North end of the chain-pier will rest on Denmark-hill, and its southern on the English Channel. As it is, the metropolis pours daily its wealth into the Portus Salutis, while the latter makes a large return of health to the care-worn inhabitants of Modern Babylon. It may admit of dispute which place has the balance, or *exchange* in its favour. We are inclined to think that the Babylonians have the advantage—and for the reasons adduced some two thousand years ago by the Roman Bard.

Prestat argento, superatque fulvum
Sanitas aurum, superatque censum
Quamvis ingentem—validæque vires
Omnia prestant.

So say we;—though the money-makers and money-changers will not believe it till they have lost the invaluable blessing of health! Many a time, when the foul fiend of the fens had poured its gelid currents through our veins till our bodies shook like a lump of jelly in a bag—then parched us in an oven—and ultimately boiled us to a boullie—have we sought the pure and bracing breezes of Brighton—and never in vain—never without putting an extinguisher on the ague-monster, and dispelling the indescribable depression which his operations induce in the human fabric!!

Brighton, independent of its salubrious air, and freedom from all malarious impregnations, must always, from its proximity to London, as a sea-bathing place, command a superiority of selection for the citizens of London, while the artificial mineral waters, so successfully manufactured there, will also swell the stream of visitors to that favourite locality. Brighton is now to the vast metropolis what BAIÆ once was to the Eternal City on the seven hills.

The author of the present brochure, though long a resident in Brighton, is now a citizen of London, and cannot, therefore, be suspected of a sinister or self-interested *motive* in his observations, the whole of which, indeed, bear the impress of truth. The topography of Brighton has not been studied with any degree of minuteness by medical men, and there can be no doubt that this little pamphlet will be a welcome present to the profession, and still more so to the non-professional invalid, about to sojourn a while in the British Baiæ.

Dr. Wigan is not only well acquainted with Brighton, but with most of the favourite watering-places both in this country and on the Continent. He can therefore speak comparatively as well as positively on the subject. We must pass entirely the introduction, which offers some excellent advice to invalids resorting to the coast for the sake of health.

The town of Brighton, Dr. W. remarks, may be divided into three parts—"The centre portion, extending from Cannon Place on the west, to the New Steyne, or even to Rock Gardens, on the east; and the two extremities, to Adelaide Terrace on the west, and to Arundel Terrace, Kemp Town, on the east, each inclusive;" or, in other words, the eastern, western, and centre portions.

The centre portion, being occupied by the permanent inhabitants, is most frequented, and indeed most recommended by the faculty in London. It differs very little, however, from an inland town in a low situation, and has nothing *bracing* in its atmosphere. Much annoyance is there experienced by the numerous steam-engines belonging to the baths and breweries, and the chimnies of private houses in the eastern and western portions, according to the direction of the wind, which blows from one or other of these quarters eight months in the year—from the north or south for the remainder.

"When the wind is due north the atmosphere at all seasons is beautifully transparent, and the whole town as brilliant as Genoa. In ordinary weather, although each extremity of the town is extremely clear, the middle portion has a mist (at least) which prevents the free ascent of the smoke, and the inhabitants of one end of the town can scarcely ever see the other extremity—each generally supposing the other end to be enveloped in smoke, when, in fact, it is as clear as his own portion.

"The centre of the town has been so effectually drained, and there is so much care employed to remove nuisances, that it is not objectionable to those in health, nor, indeed, to more than one class of invalids, and it presents the advantage of baths, libraries, shops, riding-schools, fencing-rooms, billiards, and other sources of health or recreation.

"To individuals without families, and who are unable to have a permanent conveyance, and to those who resort to the baths for *maladies requiring surgical aid only*, or who merely come to Brighton to escape the turmoil of business, or the thick atmosphere of London, this part of the town is well adapted; but such as have their families with them, and can afford the larger and better built houses of the East and West, should not subject themselves to the disadvantages and annoyances inseparable from a low situation in the centre of a large town.

"The eastern portion of Brighton is elevated considerably above the sea, and, except when the wind is violent, escapes in a great degree the admixture of saline particles with the atmosphere; not chemically, but mechanically suspended therein. It has a chalky soil, through which the rain immediately percolates, and which permits no moisture to remain on the surface. The houses, too, are of modern erection, and possess many of the comforts and conveniences which have only lately been brought into general use, and the air is decidedly 'bracing.'

"The western portion, again, (at least, that part of it which abuts on the sea,) has a clayey soil, and a mild and soft climate. The clay has, however, been so largely excavated for building, and for bricks, and the immense lawn in front of the sea is so entirely composed of artificial soil, covering a bed of rounded pebbles, and the country further to the west in the direction of the prevalent winds is so entirely gravel, that it escapes in a great degree the annoyances which the original composition of the soil would have seemed to indicate. *I consider that portion of the western division of the town which is north (and, perhaps, inclusive) of the western road; the upper part of Montpellier Road; extending to the New Vicarage, and the house called the Temple, and round to the Poor House, to be by far the most salubrious portion of the whole town.* There are, indeed, very few, if any, maladies for which the air of Brighton is advisable that will not be more favourably placed here than elsewhere." 29.

Dr. W. is convinced that, "*in all cases whatever*, the sea air is more salubrious at the distance of five or six hundred yards from the edge of the water than close to it."

"Those houses in the front line, in the middle portion of the town, which are let into lodgings, are mostly small and ill-built; small low rooms in front; still smaller, close, and dark bed-rooms behind. Of course there are some exceptions. The sun in winter is so cheering and attractive that visitors are glad to have the most of it, and do not consider some disagreeable collateral circumstances. In summer, *when the sun is high*, there is no reflexion from the water to interrupt the view in a southern aspect, but in winter, *when the sun is low*, it gives nothing but an irritating glare from the surface of the sea, which prevents anything from being seen, and makes it necessary to keep down the blinds till almost sun-set. When there is no winter sun, and the weather is misty, the sea presents generally the aspect of a *dull grey wall*, an object anything but agreeable.

"From the middle of July to the middle of October, invalids may locate themselves as close to the water as they please—or on it. At other seasons let them keep at a short distance, and if they have the power to choose, select a house with an eastern aspect. This, by the by, from the structure of the town, will generally be south-eastern; take it altogether, the best 'exposition' of all." 32.

From the middle of March to the middle of May—Dr. W. advises invalids to reside—as far as they please from Brighton. In the above period, the heavy fogs in the Channel are floated on the tides to the land, where they adhere too strongly to be floated off again to sea on the ebb. The atmosphere is so unequally heated, that portions, at the distance of a few yards only, will vary five or six degrees in temperature—sometimes double that, especially in calm weather and sheltered localities. Upon these states a bitter cold East wind will often supervene, rendering that part of the coast very objectionable.

"Indeed, in March and April it is no uncommon thing to see an attack of erysipelas from imprudent exposure in what appears splendid weather; and sometimes a severe attack of jaundice, very difficult of cure. Even when the latter does not take place, there is a *tendency to it,* which produces great debility and discomfort. *I know no malady whatever,* NOT EVEN GLANDULAR DISEASE, *which is benefited by sea-air thus early in the season.* Even persons in health come in from a walk or ride unrefreshed, fatigued, and suffering from headache; and the kidneys act in such excess as to give almost the appearance and almost the effect of diabetes. This symptom is, indeed, a frequent effect of sea-air at all seasons, and, when it does not subside spontaneously, requires attention, or it will defeat every attempt to re-establish vigour." 34.

Our author recommends people who are obliged to reside in Brighton, to stay in doors till after an early and nutritious dinner, and to burn wood instead of coal—at any rate to let a portion of the fuel be wood, and to keep a clear fire and a strong draught in the chimney, thus escaping the strong gases evolved from the coal, when people are compelled to stay indoors.

The months of June and July are not only the most delightful period of the year; but that in which the air of the sea at Brighton has the most energetic influence in the cure of disease. The sea-fogs are over—the air has acquired a uniformity of temperature, though not too warm to prevent exercise—the pathways and roads through the corn-fields afford most pleasant rides and walks—the downs are in perfection—and open vehicles may be had on very moderate terms. The fields, East of the Windmill, overlooking the beautifully wooded village of Preston, is one of the most agreeable spots on all this coast.

"At whatever season, I recommend, that when the wind blows with violence from the sea, the accustomed promenade should be directed towards the country. A quantity of fine sand from the beach, so minutely, impalpable as to be almost a fluid, is suspended in the air, and necessarily inhaled: and though it has none but mechanical effects, it is sufficient to produce a sense of oppression and weariness; and to tracheal disease and irritable lungs is obviously injurious.

"Many great towns are partially surrounded by gardener's grounds or highly cultivated fields. This is remarkably the case in some of the suburbs of London, where a constant succession of fresh manure is used, to force two, three, or even four crops in the year. This animal and vegetable filth, in a state of decomposition, is spread over the land sometimes twice a year, contaminating the atmosphere to a great distance around, and produces extensive disease. It is strange that the public should be so very indifferent to this,—that they should inhabit, by choice, houses built in the midst of this sea of putrefaction, when, if an offensive odour arise from a drain or stagnant puddle in the town, they are up in arms to remove the nuisance. I knew and attended in succession five families in a single row of houses in a locality of this description, which I must not name more specifically. All of these families were ultimately compelled to quit the neighbourhood, from a succession of anomalous ailments and frequent fevers. I believe, indeed, that the fevers so common among farmers and farm-labourers often arise from this cause.

"Now, from all these and similar causes of indisposition, Brighton is entirely exempt. Its supply of vegetables is brought from a distance, and the Downs, with their dry soil and elastic turf, equal to the greensward of a fine lawn—the Downs, which surround the town, furnish the purest and most exhilarating air in the world. Never on the Alps, the Appenines, or the Jura have I felt so intensely, so *exultingly, the abstract pleasure of mere animal existence,* as on the Downs in the neighbourhood of Brighton. No manure, no decomposition on the surface, because no humidity will remain there,—at such a distance, or such an elevation above the sea, that all which is insalubrious in the air has been deposited before it reaches them. A canter over the Downs in a fine day produces the feelings of the Arab in the desert: the breathing deep and complete, and every air-cell of the lungs fully opened and performing its duty. Eat and drink whatever you please, and as much as you please, if you can take abundant exercise on the Downs. I believe, indeed, that an occasional excess in the pleasures of the table is harmless, and even beneficial, when you have such a rectifier as this. I speak, of course, here chiefly of such as have been suffering from confinement to a town, and insufficient exercise. There are very few of the diseases (not organic) to which such persons are liable that will not yield to this kind of exercise—to fencing and to tennis. *An occasional excess, and an occasional fast, call into action some of the most valuable functions of the stomach left in abeyance by those who practise extreme regularity.*" 40.

Dr. Wigan observes that, according to his experience, "ALL CONGESTIVE DISEASES (with very few exceptions) ARE AGGRAVATED BY A RESIDENCE AT BRIGHTON."

"By 'congestive,' I mean such diseases as are caused or accompanied by local accumulation of blood, principally venous, circulating languidly in any of the organs of the body, and unattended with inflammation.

"It would not be difficult to give a plausible explanation of the above, but I prefer to state the simple fact, (of which my conviction is absolute,) and leave each practitioner to form his own opinion.

"For a long time I lent a very deaf ear to this very disagreeable conviction, but in the end felt it a matter of conscience to send all such persons, not only away from Brighton, but from all influence of sea-air. A man will be rarely wrong in acting boldly on convictions which are decidedly opposed to his own interest; and I rest absolutely and entirely on this." 41.

Inflammatory dyspepsia is another of the ailments for which Brighton is objectionable—and generally in diseases of the kidneys and bladder unattended by *albuminous secretion,* which, Dr. W. says, is checked by the air of the place. The localities differ remarkably—some being beneficial—some noxious to disorders. Even that delightful promenade—the chain-pier—he thinks, produces so much mischief in the early part of the year, that the medical men of the town ought to pay for its wear and tear, on account of the practice which it brings them! The sheltered walk, under the cliff which leads to it, affords a safe retreat from the bitter cold north-east winds. The reflected heat from the Cliffs gives quite the feeling of Summer—and this shelter and warmth will even extend to half the length of the pier itself. At a certain point, however, you lose the protection of the Cliff, and pass, at once, from a warm to a cold climate.

"A very valuable auxiliary in the treatment of disease at Brighton is THE GERMAN SPA; where almost all the celebrated waters of England, of the Continent, and even of America are prepared in absolute perfection by the consummate chemist who superintends the establishment. Although, however, some of the cures performed by these waters seem little less than miraculous, I am inclined to attribute a large portion of them to the *early rising*, the *active exercise*, the *abstinence*, the *air of the Downs*, the *entire change of habits*, and, above all, to that *thorough 'cleaning' of the internal coats of the stomach*, which accompanies their administration. The combined effect is that of establishing a *completely new sanguification*. That is, it creates a perfectly new mass of healthy blood." 49.

The natural water of Brighton is excellent, and now abundantly supplied by water-companies, whose works are situated on the Lewes road. The water filters through several hundred feet of chalk. The well-water in the town, though naturally pure, is too often contaminated by the cesspools. Baths are numerous enough here, and although douches are procurable at all of them, they are very seldom demanded—from the great ignorance respecting it which prevails among the profession in this country. In the following passage we quite agree. It is the doctrine we have been preaching for nearly twenty years.

"On the subject of general bathing, information can be obtained from so many sources that it is quite unnecessary to dwell upon it; but bathing in the open sea by invalids, and especially by young females, requires a great deal of caution. The chance of benefit rarely equals the risk, and the mischief done every season by *premature, excessive*, or *improper*, bathing is enormous. It is really frightful to see the consequences which sometimes arise in young females from indiscriminate bathing in the sea. Many a case of chlorosis have I seen changed into phthisis by this dangerous practice. No young female ought to bathe in the sea, unless under the clearest medical instructions of a man on the spot. All, and more than all the benefits may be obtained by modifications of house bathing, and by resources, which practice and observation have made familiar to medical men." 53.

One of the chief annoyances at Brighton is the extreme violence of the wind—especially when it is Easterly, for then the pungency is beyond expression. This wind is uniformly dry, and has much fine sand suspended in it. "*On such occasions stay at home*, unless you are well enough to resort to fencing, tennis, or such like amusements." (55.) Even the respirator is no safeguard against the fine particles of dust which penetrate readily through its bars and apertures. The complicated structures in the nose resemble not a little the respirator in warming the air, and arresting the influx of sand or dust. When people are obliged to go out in such winds as those described, they should endeavour, as much as possible, to breathe through the nose. In strong exercise, however, they cannot do so, and then the respirator is of some use.

We must now conclude. We have afforded sufficient samples of this little work to prove its utility: and we would advise all invalids going to Brighton to take it with them—and all medical men who are in the habit of sending patients to that place, to look into the pamphlet, lest they injure, instead of benefitting their clients.

